# Systematic review and meta-analysis of prognostic microRNA biomarkers for survival outcome in nasopharyngeal carcinoma

**DOI:** 10.1371/journal.pone.0209760

**Published:** 2019-02-08

**Authors:** Shanthi Sabarimurugan, Chellan Kumarasamy, Siddhartha Baxi, Arikketh Devi, Rama Jayaraj

**Affiliations:** 1 School of Biosciences and Technology, Vellore Institute of Technology (VIT), Vellore, Tamil Nadu, India; 2 University of Adelaide, North Terrace Campus, Adelaide South Australia, Australia; 3 Genesis Cancer Care Centre, Bunbury, Western Australia; 4 Department of Genetic Engineering, SRM Institute of Science and Technology, Kattangulathur, Tamilnadu, India; 5 Clinical Sciences, College of Health and Human Sciences, Charles Darwin University, Ellengowan Drive, Casuarina, Northern Territory, Australia; Osaka General Medical Center, JAPAN

## Abstract

**Background:**

Nasopharyngeal cancer (NPC), despite being one of the most malignant head and neck carcinomas (HNC), lacks comprehensive prognostic biomarkers that predict patient survival. Therefore, this systematic review and meta-analysis is aimed to evaluate the potential prognostic value of miRNAs as prognostic biomarkers in NPC.

**Methods:**

PRISMA guidelines were used to conduct this systematic review and meta-analysis study. Permutations of multiple “search key-words” were used for the search strategy, which was limited to articles published between January 2012 and March 2018. The retrieved articles were meticulously searched with multi-level screening by two reviewers and confirmed by other reviewers. Meta-analysis was performed using Hazard Ratios (HR) and associated 95% Confidence Interval (CI) of survival obtained from previously published studies. Publication bias was assessed by Egger’s bias indicator test and funnel plot symmetry.

**Results:**

A total of 5069 patients across 21 studies were considered eligible for inclusion in the systematic review, with 65 miRNAs being evaluated in the subsequent meta-analysis. Most articles included in this study originated from China and one study from North Africa. The forest plot was generated using cumulated survival data, resulting in a pooled HR value of 1.196 (95% CI: 0.893–1.601) indicating that the upregulated miRNAs increased the likelihood of death of NPC patients by 19%.

**Conclusion:**

To our knowledge, this is the first meta-analysis that examines the prognostic effectiveness of miRNAs as biomarkers in NPC patients. We noted that the combined effect estimate of HR across multiple studies indicated that increased miRNA expression in NPC potentially leads to poor overall survival. However, further large-scale prospective studies on the clinical significance of the miRNAs, with sizable cohorts are necessary in order to obtain conclusive results.

## Introduction

Nasopharyngeal carcinoma (NPC) is a malignant epithelial Head and Neck Carcinoma (HNC) [1,2] and is primarily prevalent in the Asian population, with 40% of the 84,000 new cases globally, being reported in China, Malaysia, Singapore, Indonesia, Vietnam and Brunei [3–5]. The molecular landscape of NPC is defined by an array of genetic and epigenetic variations, which, in most NPC cases express a malignant phenotype, when combined with dormant Epstein-Barr Virus (EBV) infection [6,7]. Advances in radiotherapy and comprehensive chemotherapy strategies have significantly improved clinical outcome of the patients with primary NPC. However, despite these developments in the treatment strategies for NPC in recent years, it is still considered as an aggressive disease due to its invasive nature, delayed diagnosis, relatively poor prognosis and overall patient survival [8].

MicroRNAs (miRNAs) are small non-coding RNAs, which may also act as oncogenes by influencing multiple cancer mechanisms, including metastasis [9,10]**. Micro**RNAs play an essential role in cancer either as tumour suppressors/repressors or promoters in many malignancies including NPC, therefore giving miRNAs potential prognostic utility [5,11]. Identification of miRNAs may function as therapeutic targets and provide new avenues to treat NPC [12]. Furthermore, EBV encoded miRNA has been shown to play a complementary role to the viral proteins which are expressed in malignant cells of the NPC, which promote survival and proliferation of NPC cells, leading to the evasion of host immune responses [6].

### Rationale

#### The importance of the issue

Many studies have investigated and reported the prognostic significance of miRNAs in multiple types of HNC, including oropharyngeal carcinoma and laryngeal squamous cell carcinoma through narrative reviews and systematic reviews and meta-analyses [5,11,13]. Although previous studies have conducted analysis regarding the relevant anatomical sub-site of HNC, there is still a knowledge gap regarding the prognostic impact of miRNAs in NPC. This knowledge could potentially be valuable for head and neck health care. Although circulating EBV PCR in blood before and after treatment showed some prognostic capability, the results remain inconsistent.

A few reviews have also highlighted the deregulated expression of miRNAs and their association with the progression of NPC and subsequently, its prognosis [5,11,14,15]. Findings from previous studies have also shown that the down-regulation of miRNAs can be associated with enhanced survival of NPC patients [16,17]. A previous meta-analysis has performed miRNA target prediction and pathway enrichment analysis to identify the functional genes involved in the meta-signature regulation of NPC. However, the authors only explored the possible pathways of NPC pathogenesis and signalling pathways and did not discuss the relationship between the miRNAs expression and patients survival [13]. While the aforementioned narrative reviews and systematic reviews and meta-analyses indicate possible diagnostic, prognostic and therapeutic use of miRNAs in other subtypes of HNC, quantitative and qualitative evaluation of miRNAs on patient survival in NPC through a systematic review and meta-analysis has not yet been conducted. As there are no approved molecular biomarkers routinely available in clinical practices as promising prognosticators in NPC and HNC, this study aims to ameliorate this deficit.

#### How will the study address this issue?

To address the significance of miRNAs as potential prognosticators in NPC, we have undertaken a systematic review and meta-analysis which provides a framework for reporting the impact of miRNA expression on patients survival and identifying the pooled effect size across all NPC prognostic studies. Obtaining a summary estimate of the relationship between increasing values of miRNAs expression and risk of death via pooling the hazard ratio across all selected studies would provide for a better understanding of the survival outcome of NPC patients. This is the first study to evaluate the qualitative and quantitative analysis of published studies on NPC prognostic research, which could be helpful to investigate both, the clinical and the biological aspects of the of the disease at the molecular level.

#### How will it help?

Given that current pathological prognostic biomarkers for NPC are unreliable, this study focuses on identifying miRNAs that have a prognostic and therapeutic utility in NPC. This study will also have clinical implications and in the long term, may help clinicians in treatment decision-making and better post-treatment care in NPC as well as improving clinical outcomes. This study should also assist in directing future clinical research and development in the field of NPC prognostication.

## Methods

PROSPERO registration number: CRD42018083945

### Search strategy

**Fig 1:**
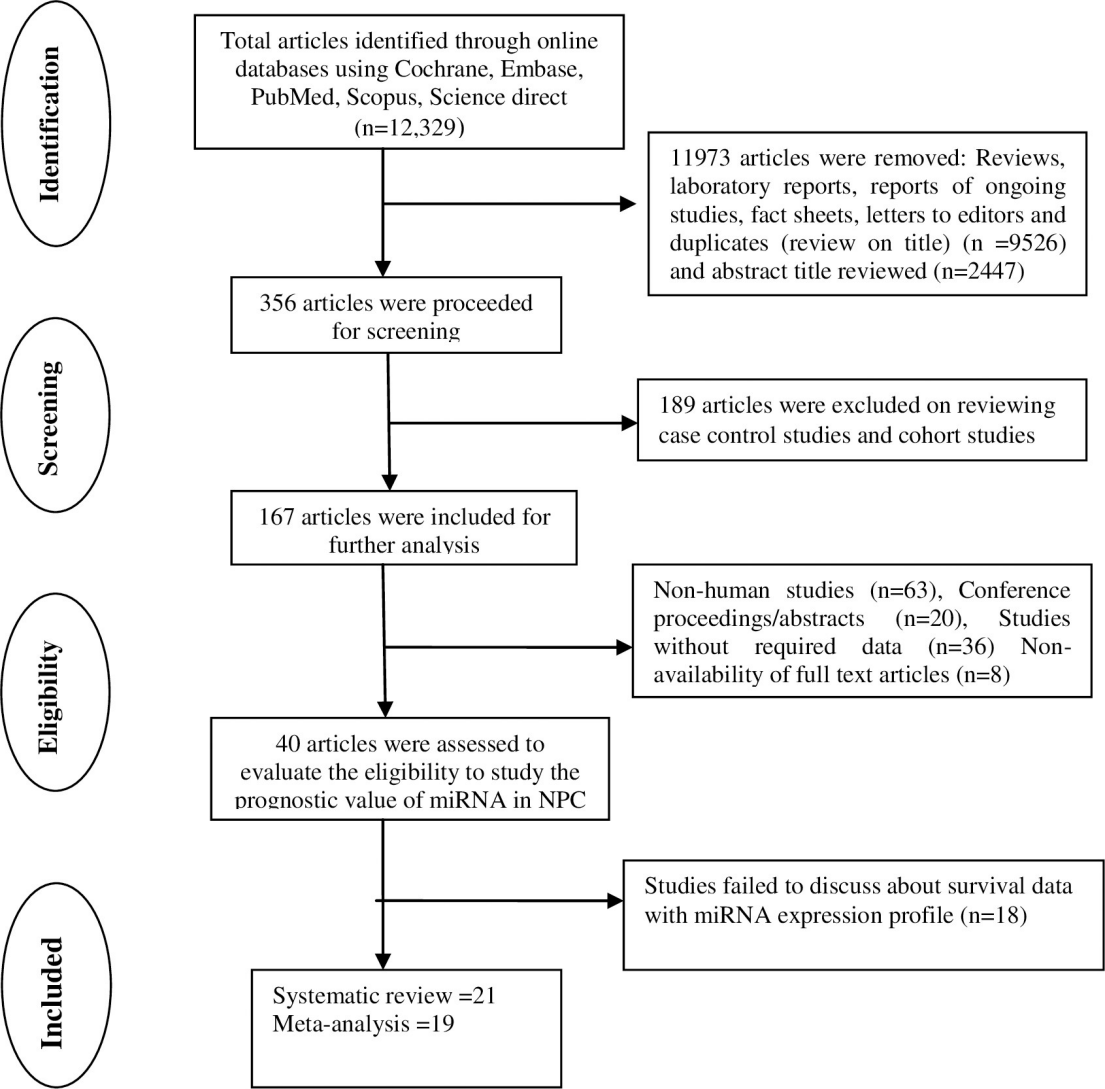
Flowchart for study selection and data acquisition.

The studies were explored using Medical Subjective Heading (MeSH) search terms. The key-terms (Table 1) also included all abbreviations, synonyms and subsets utilised as part of our search strategy. All relevant studies from 2012 to 2018 were searched in the Cochrane, EMBASE, PubMed, Science Direct and Scopus databases. Two reviewers examined the titles and abstracts independently to identify all eligible studies. The reference lists of the selected articles were also reviewed to locate further articles to increase the robustness of the search. Any disagreements between the two reviewers were resolved through mutual discussion, following consultation with a third reviewer. A diagrammatic representation detailing the selection process has been presented (Fig 1). The retrieved studies were assessed as per the criteria specified in the Preferred Reporting Items for Systematic Review and Meta-Analysis (PRISMA) guidelines for systematic review and meta-analysis [18].

**Table 1:** MeSH search terms utilised in the search strategy.

1	“Nasopharyngeal carcinoma” [Topic] OR “Pharynx carcinoma” [Topic] AND “miRNA” [Topic]
2	“Nose and throat cancer” [Topic] AND “Epidemiology” [Topic]
3	“Prognosis” [Topic] OR “Survival” [Topic]
4	“Upregulation” [Topic] OR “Downregulation in NPC” [Topic] OR “Differential expression” [Topic] or “Deregulated miRNAs” [Topic]
5	“Meta-analysis study” [Topic] OR “Systematic review” [Topic] AND “NPC” [Topic]
6	“Follow up studies.” [Topic] OR “miRNA” [Topic]
7	“Prognosis” [Topic] OR “Survival outcome” [Topic] OR “Hazard Ratio” [Topic]
8	Treatment of NPC [Topic] OR “Prevalence” [Topic] AND “Worldwide” [Topic]
9	“Incidence” [Topic] AND “Risk factors” [Topic]
10	“Epstein bar virus” [Topic]

### Selection criteria

The studies fulfilling the following criteria were included in the systematic review.

*Inclusion criteria*:

1. Studies that discussed prognosis of miRNAs in NPC patients.
2. Studies which were published from 2012 to 2018.
3. Studies that published the miRNA expression profile, and clinicopathological parameters.
4. Studies that examined the NPC patients’ survival and presented associated Hazard Ratio (HR), with 95% confidence interval (CI) for OS, DFS or DSS as numerical data or KM curves.
5. Studies that conform to the PRISMA guidelines for systematic review and meta-analysis.

The following types of studies were excluded

*Exclusion Criteria*:

1. Studies not published in English
2. Studies using duplicated data.
3. Narrative reviews, fact sheets, case-control studies, cohort studies, intervention studies, letters to editors, laboratory studies, nonhuman studies, and reports.
4. Unpublished materials, uninterpretable data, conference proceedings or thesis.
5. Studies reporting prognosis results for sample size <10

### Data extraction and management

We retrieved information about NPC patients clinical and treatment parameters presented in the published studies. Information about the type of sampling method was also collected. The retrieved data were recorded in a custom ‘Data Extraction Form’ generated using Microsoft Excel for further evaluation of study quality and data synthesis. The review team also extracted the following data items from the studies; such as author names, year of publication, study period, geographic origin, number of patients, number of clinical samples, source of the clinical samples, histological type, lymph node metastasis/distant metastasis, miRNAs studied, miRNAs detection platform, miRNA expression profiles, follow-up period, NPC patients survival data (HR and 95% CI), overall survival (OS), progression-free survival (PFS) and recurrence-free survival (RFS) and miRNA dysregulation studied by individual study.

### Quality assessment

The methodological quality was assessed by a quality assessment template based on the National Heart, Lung and Blood Institute (NHLBI) for Systematic Reviews and Meta-analysis [19]. This assessment template was used to evaluate the selected full-text studies which were considered eligible for systematic review (rated as good, satisfactory and bad).

### Meta-analysis and assessment of heterogeneity

Comprehensive Meta-Analysis (CMA) software (version 3.3.070, USA) was used to analyse the data and perform the meta-analysis. Forest plots were generated using NPC patient survival data (HR and 95% CI) from the selected studies. The mean effect estimate of HR is more frequently calculated in meta-analysis compared to the statistical significant and sample size of included studies [20]. Pooled estimates of HR were estimated by a random-effects model due to high between-study heterogeneity. Heterogeneity was assessed using Higgin’s I^2^ statistic and Cochran’s Q-test [21]. Tau-sqared statictics as a part of the statistical analysis performed in the study and is the estimated variation between the effects for test accuracy observed in different study [22,23].Studies that reported the survival data in the form of Kaplan Meier curves were used after calculating the HR and 95% CI of survival from the KM curves. Meta-analysis of the prognostic factor study frequently estimate multiple cut off point and methods of measurements [24]. Q test will be estimated at a p value of under less 0.001 indicating statistical significance [25]. The subgroup analysis was performed with the subgroups as miRNAs most frequently represented in the selected pool of studies.

### Publication bias

Inverted funnel plot symmetry was used to observe for the presence of any publication bias. We also examined our included studies using classical bias indicators as Harbord-Egger’s Test of the intercept, ‘Orwin’s and classic fail-safe N test,’ Begg and Mazumdar Rank correlation test,’ Duval and ‘Tweedie’s trim and fill’ calculation. The detailed description of these tests. are as follows.

#### Classic fail-safe N and the Orwin fail-safe N Test

Both of the fail-safe N (The Classic failsafe–N and Orwin fail-N safe test) addressed the possibility to interpret true or false whether studies were missing from the analysis and that these studies if included in the analysis, would shift the effect size toward the null [26]. Both Orwin fail were applied to estimate studies that are missing from systematic review and meta-analysis [27].

#### Begg and Mazumdar rank correlation test

There would be an inverse correlation between study size and effect size in regardless of their prognostic effects with small studies as well as large studies [28]. Therefore, we processed the rank order correlation (Kendall's tau b) between the prognostic effect and the standard error (which is driven primarily by sample size).

#### Egger's test of the intercept

Egger’s test suggested the same bias by normalised effect estimate (estimate divided by its standard error) against precision (reciprocal of the standard error of the estimate). In this equation, the size of the prognostic effect was captured by the slope of the regression line (B1) while bias was captured by the intercept (B0) [29].

#### Duval and Tweedie's trim and fill

This method initially trimmed the asymmetric studies from the right-hand side to locate the unbiased effect (in an iterative procedure), and then filled the plot by re-inserting the trimmed studies on the right as well as their imputed counterparts to the left the mean effect [30]^.^

## Results

### Study selection

Fig 1. represents the schematic presentation of the selected studies. The literature search revealed a total of 12,329 publications, out of which 9526 were excluded after a preliminary screening of the publications based on their titles, and 2447 were removed upon examination of abstracts, leaving only 356 studies that were considered relevant. Further applying the MeSH search terms on the abstracts, and selecting only the studies to which access to the full-texts was available, resulted in the recovery of 167 studies. From these, another 127 articles were excluded as they were out of the scope of our systematic review and meta-analysis, such as, studies not focusing on human samples (n = 63), conference abstracts (n = 20), the absence of full-text articles (n = 8) and studies that did not analyse miRNA expression in NPC (n = 36). Double verification of existing reviews and meta-analyses’ reference lists revealed no further relevant articles [1,5,8,11,14,15,31].

Upon careful review of 40 full-text articles against the predefined inclusion criteria, six did not provide HR values, 12 did not examine NPC patients survival with regards to miRNA expression, leaving only 22 studies discussing the prognostic significance of miRNAs in NPC patients. One study has been retracted form the respective Journal and hence we didn’t include that in the study. So finally, 21 studies [10,12, 32–39, 16, 40–48, 17] were included in this systematic review and meta-analysis. Among these, only 14 studies were found to report the HR, and 95% CI values with the other seven articles presenting the survival data in the form of Kaplan Meier curves alone which are mentioned in detailed within Table 2. The Hazard Ratio and 95% CI values were extracted from Kaplan Meier curves of six articles, with one remaining article having insufficient data. A final total of 20 articles were selected for the meta-analysis, as the HR and CI values of one study [43] could not be retrieved.

**Table 2:** Study characteristics included in the systematic review and meta-analysis.

S.No	Study	Population	Study period	Gender	Sample size	Source of sample	Platform	Followup period	miRNA studied	Histological type	Lymph node metastasis/ Distant metastasis	Cancer type/Subtype	Endpoints	HR value	miRNA dysregulation
1	Allaya et al., 2015	North Africa	NR^a^	NP^a^	43	Tissue	Conventional PCR	NP^a^	miR10b	NKC^b^, UNCT^c^	N0-N3/M0, M1	T1, T2, T3 & T4	OS^h^	KM^n^ Curve alone	Upregulated
2	Chen et al., 2016	China	NR^a^	M-58/F-23	81	Tissue	qRT-PCR	NP^a^	miR17-5p	NP^a^	N0-N3/M0,M1	T1, T2, T3 & T4	OS	KM^n^ Curve alone	Upregulated
	He et al., 2017	China	NR^a^	M-54/F-23	77	Serum	qRT-PCR	5 year	miR21	NR^a^	N0-N3/studied but not mentioned	T1, T2, T3 & T4	OS^h^, DFS^i^	KM^n^ Curve alone	Upregulated
5	Huang et al., 2016	China	NR^a^	N/A	62	Tissue	qRT-PCR	NR^a^	miR19b-3p	Neoplasm	N0-N3	T1, T2, T3 & T4	OS^h^	Provided	Upregulated
6	Liang et al., 2016	China	NR^a^	M-40/F-34	74	Serum	qRT-PCR	5 year	miR663	NR^a^	Studied but not mentioned exact stage	T1, T2, T3 & T4	OS^h^, RFS^l^	Provided	Upregulated
7	Liu et al., 2013 a)	China	January 2003 and February 2006,	M-163/F-54	237	Tissue	qRT-PCR	62.5 months	miR29c	Locoregional failure	N0-N3/studied but not mentioned	T1, T2, T3 & T4	OS^h^, DFS^i^, DMFS^m^	Provided	20 Samples: miRNA expression alone -Downregulated: 217 samples expressed miRNA with protein—Upregulated
8	Liu et al., 2013b)	China	January 2003 and February 2006.	M-206/F-74	300	Tissue	qRT-PCR	63.9 months	miR451	NR^a^	N0-N3	T1, T2, T3 & T4	OS^h^, DFS^i^	Provided	Downregulated
9	Liu et al., 2014	China	January 2001 and December 2006.	M-101/F-27	512	Serum	microarray analysis/qRT-PCR	78.4 months	miR22, miR572, miR638 &miR1234	Keratinizing (SC^e^)& nonkeratinizing	N2-N3	T3-T4	OS^h^, DMFS^m^	Provided	Upregulated
10	Liu et al., 2012	China	312 samples: Jan 16, 2003 and Feb 25,2006/ 153 samples: April 1, 2002, and May 22, 2008	N/A	468	Tissue	microarray analysis/qRT-PCR	62.1 months	41 miRNAs	Keratinizing (SC^e^), undifferentiated keratinizing & nonkeratinizing	N0-N3/studied but not mentioned	T1, T2, T3 & T4	DFS^i^/DMFS^m^	Provided	Upregulated
11	Luo et al., 2013	China	NR^a^	M-127/F-41	12	Tissue	microarray analysis/qRT-PCR	NR^a^	miR18a	NR^a^	Studied but not mentioned exact stage	NR^a^	OS^h^	Provided	Upregulated
12	Ma et al., 2014	China	NR^a^	M-136/F-139	275	Tissue	qRT-PCR	NR^a^	miR204	NR^a^	N0-N3/M0,M1	T1, T2, T3 & T4	OS^h^	KM^n^ Curve alone	Downregulated
13	Wang et al., 2014	China	January 2009 and April 2009	M-69/F-31	100	Plasma	qPCR with microRNA assay	86 months	miR483-5p, miR103, miR 29a and let -7c	KSCC^d^, NKDC^g^, NKUC^f^	NR^a^	NR^a^	OS^h^/PFS^k^	Provided	miR483-5p and miR103—down regulated; miR29a, let-7c- Upregulated
14	Yu et al., 2013	China	NR^a^	M-117/F-56	213	Tissue	qRT-PCR	NR^a^	miR18b	NR^a^	N0-N3/studied but not mentioned	T1, T2, T3 & T4	OS^h^	Provided	Down-regulated
15	Yu et al., 2015	China	NR^a^	M-46/F-40	86	Plasma	qRT-PCR	5 year	miR744	NR^a^	N0-N3/M0,M1	T1, T2, T3 & T4	OS^h^	Provided	Down regulated
16	Zeng et al., 2012	China	NR^a^	M-98/F-62	303	Serum	TaqMan Low-Density Array & qRT-PCR	NR^a^	miR17, miR20a, miR29c & miR223	SC^e^	NR^a^	T1, T2, T3 & T4	OS^h^, DFS^i^	KM^n^ Curve alone	Upregulated
17	Zhang et al., 2016	China	January 2006 to December 2009	M-63/F-23	86	Tissue	qRT-PCR	NR^a^	miR92a	NR^a^	Not lymph node but Distant metastasis studied	T1, T2, T3 & T4	OS^h^, DFS^i^	Provided	Upregulated
18	Zhang et al., 2017	China	July 2006 to October 2015	M-36/F-18	54	Tissue	qRT-PCR	4–62 months	miR324-3p	SC^e^	Studied but not mentioned exact stage	T1, T2, T3 & T4	OS^h^	KM^n^ Curve alone	Down-regulated
19	Zhao et al., 2016	China	NR^a^	M-99/F-43	142	Tissue	qRT-PCR	NR^a^	miR3188	NR^a^	N0-N3/studied but not reported	T1, T2, T3 & T4	OS^h^	KM^n^ Curve alone	Down-regulated
20	Zhao et al., 2017	China	Jan2011 to Dec 2016.	M-67/F-22	89	Tissue	qRT-PCR	5 year	miR92b	NKC^b^, KSCC^d^	Studied but not mentioned exact stage	T1, T2, T3 & T4	OS^h^, DFS^i^	Provided	Down regulated
21	Zhen et al., 2013	China	NR^a^	M-128/F-62	190	Tissue	qRT-PCR	4 to 126 months	miR184	NR^a^	NR^a^	NR^a^	OS^h^	Provided	Down regulated
22	Zheng et al., 2013	China	Between 2000 and 2009 and were followed up until 2012	M-1182 / F-483	1665	Tissue	microarray analysis/qRT-PCR	every three months by telephone for the first three years, every six months in years 4 and 5, and annually after that.	miR608	Differentiated and undifferentiated carcinoma	NR^a^	NR^a^	TTR^j^, OS^h^	Provided	Not provided

^**a**^Not Reported

^b^Non-keratinizing carcinoma.

^c^Undifferentiated carcinoma of Nasopharyngeal Type.

^d^KSCC-Keratinizing Squamous cell Carcinoma.

^e^SC-Squamous cell carcinoma.

^f^NKUC- Nonkeratinizing undifferentiated carcinomas.

^g^NKDC-Non keratinising differentiated carcinoma.

^h^OS-Overall Survival.

^i^DFS-Disease-free survival.

^j^TTR-Time to recurrence.

^k^PFS-progression free survival.

^l^RFS-Recurrence free survival.

^m^DMFS-Distant metastasis-free survival.

^n^Kaplan Meier

### Study characteristics

Table 2 describes the main characteristics of the 21 studies, evaluating a total of 70 miRNA prognostic biomarkers which are included in the systematic review. Of these studies, 20 studies were conducted in China and one in North Africa [10] There were a total of 5069 patients across all included studies, considered for analysis with variable cohort sizes in the individual studies ranging from 12 to 1665 patients. MicroRNA expression was found to have been analysed in fresh/preserved samples of tissue (15 studies), serum (4 studies) and plasma (2 studies). Only one study [43]used TaqMan Low-density array for miRNA expression profiling whereas 20 studies examined miRNA expression using RT-PCR. Ten studies did not provide the follow-up period data, and the average follow-up periods from other studies ranged from two to 10.5 years. A total of 65 miRNAs were reported among all the studies considered for the systematic review and meta-analysis, with all studies reporting the miRNA dysregulation status except one study conducted by Zeng et al., (2013) [48] and non availability of HR value from Zeng et al., 2012 [43]. From the rest of the 65 miRNA included, 54 miRNA were found to be upregulated and 11 miRNAs were downregulated.

### Meta-analysis and survival outcome

**Fig 2:**
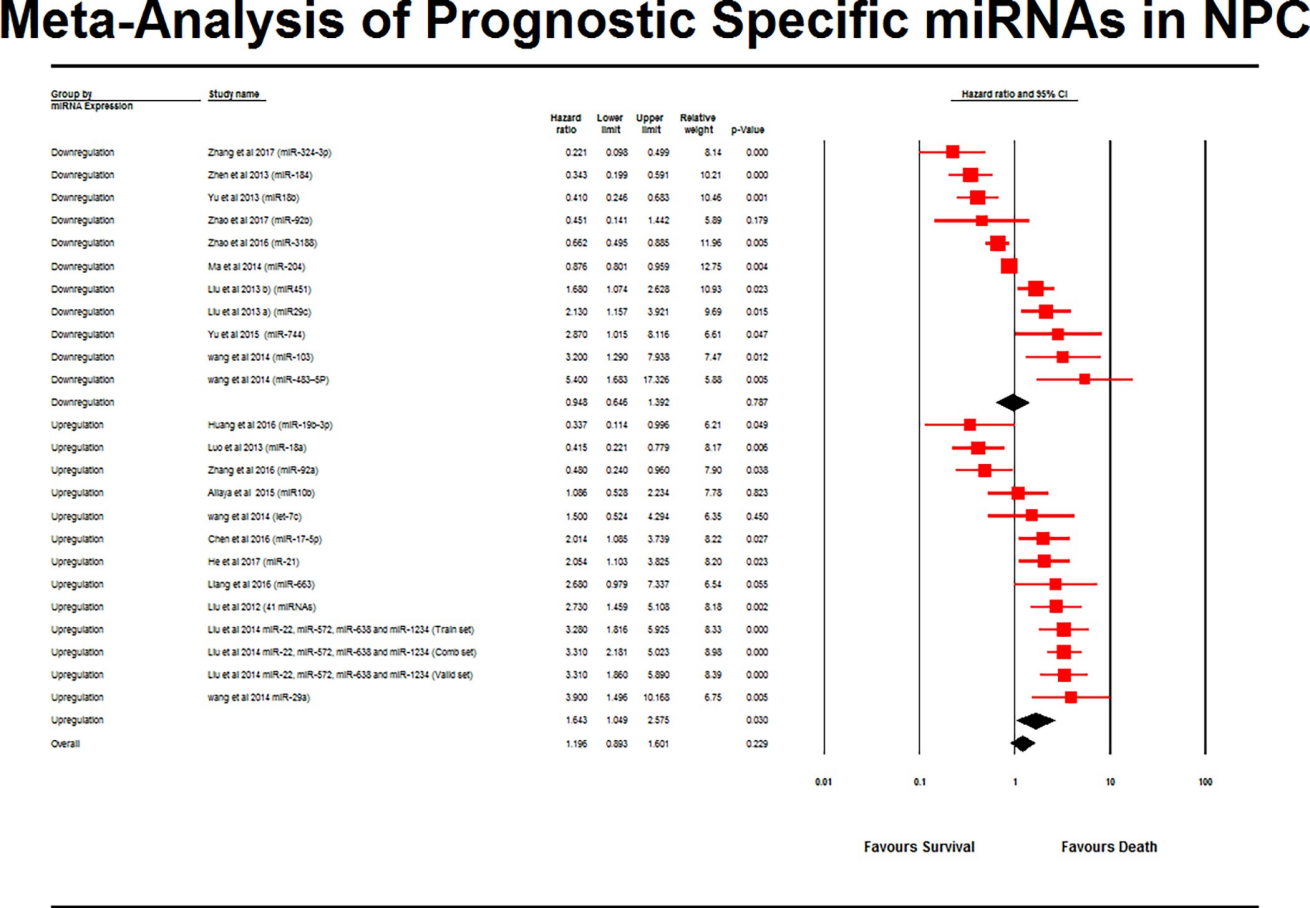
Forest plot for survival outcome of miRNAs in NPC patients. The pooled hazard ratios of HR values for NPC prognostic data were calculated and analysed using CMA software (version 3.3.070, USA). The red square represents the pooled effect estimate of survival for NPC patients randomly assigned to miRNA evaluation. The black diamond with line indicates the effect size of miRNA of the included studies with 95% confidence interval. The risk ratio of 1 suggests no difference in risk of NPC patients survival. A risk ratio > 1 indicates an increased risk of patients survival whereas a risk ratio < 1 suggests a reduced risk of patients survival. Favours A refers to better survival and B indicates worse survival.

The results of the meta-analysis were based on the survival data of 3101 NPC patients, evaluating total of 65 miRNAs as potential prognostic markers, being reported across 24 individual miRNA studies (Fig 2). Five miRNAs from the total of 70 miRNAs were not included in the meta-analysis due to a lack of miRNA expression data and an absence of HR & CI values being reported (Zeng et al. 2012 [43] (4 miRNAs) and (Zheng et al. 2013 [48]) (1 miRNA).

#### Does miRNA expression affect NPC patient’s survival?

The overall pooled effect estimate of HR was 1.196 which indicated that the upregulation of miRNAs increased the likelihood of death of NPC patients by 19%. The confidence interval for the pooled HR value was between 0.893–1.601 when assessed by the random effects model. Since the studies included in this analysis were all obtained from the currently available pool of NPC studies, the confidence interval for the hazard ratios revealed to informs us that the mean HR of any of the selected studies would fall within this range. The Z value for test null hypothesis (mean risk ratio is 1.0) was 1.202, with the corresponding *P*-value of 0.229. We can reject the null hypothesis that the risk of an event was the same in both up-regulated and down-regulated groups, and conclude that the risk of death was higher in the up-regulated group.

Out of these 65 miRNA across 24 studies, 11 miRNAs were downregulated and 54 miRNAs were upregulated. Of the 11 downregulated miRNAs in NPC, five miRNAs (miR451, miR29c, miR744, miR103 and miR483-5p) indicated poor prognosis and six miRNAs (miR324-3p, miR184, miR18b, miR92b, miR3188, miR204) were associated with a good prognosis. Conversely, elevated expression of miR19b-3p, miR18a, miR92a was correlated with a good prognosis and miR10b, let-7c, miR17-5p, miR21, miR663, miR22, miR572, miR638, miR1234 and miR29a were associated with poor patient survival, potentially indicating a poor prognosis.

#### How much does the effect size vary across studies?

The Q-statistic provides a test of the null hypothesis that all studies in the analysis share a common effect size. If all studies shared the same effect size, the expected Q would be equal to the degrees of freedom (the number of studies minus 1). The Q-value was 197.92 with 25 degrees of freedom and a p-value of 0.522. Since the observed variance falls within the range that can be attributed to sampling error, we cannot reject the null that the true effect size was the same in all studies.

While the data did not prove that the effect size varies across populations, we can assume that it does. Also, we can proceed to estimate the extent of the variation. The I^2^ statistic tells us what proportion of the observed variance reflects differences in true effect sizes rather than sampling error. Here, I^2^ was 88.380%. T^2^ is the variance of true effect sizes (in log units). Here, T^2^ was 0.451. T is the standard deviation of true effects (in log units). Here, T was 0.671.

#### Does the effect size vary by subgroup?

While the mean effect size across all studies was modest (a hazard ratio of 1.196, it’s possible that the mean hazard ratio varied by subgroup. We used the subgroup analysis to compare the effect size in studies that employed a high expression (upregulation) and low expression (downregulation) of miRNAs. The mean risk ratio in the two groups was 1.643 and 0.948 respectively. The Q value for the differences was 3.325 with 25 *df*, and *P* value equals 0.060. Therefore, there was no evidence that hazard ratios varied as result of NPC patients’ survival.

### Publication bias and sensitivity analysis

#### Funnel plot

**Fig 3:**
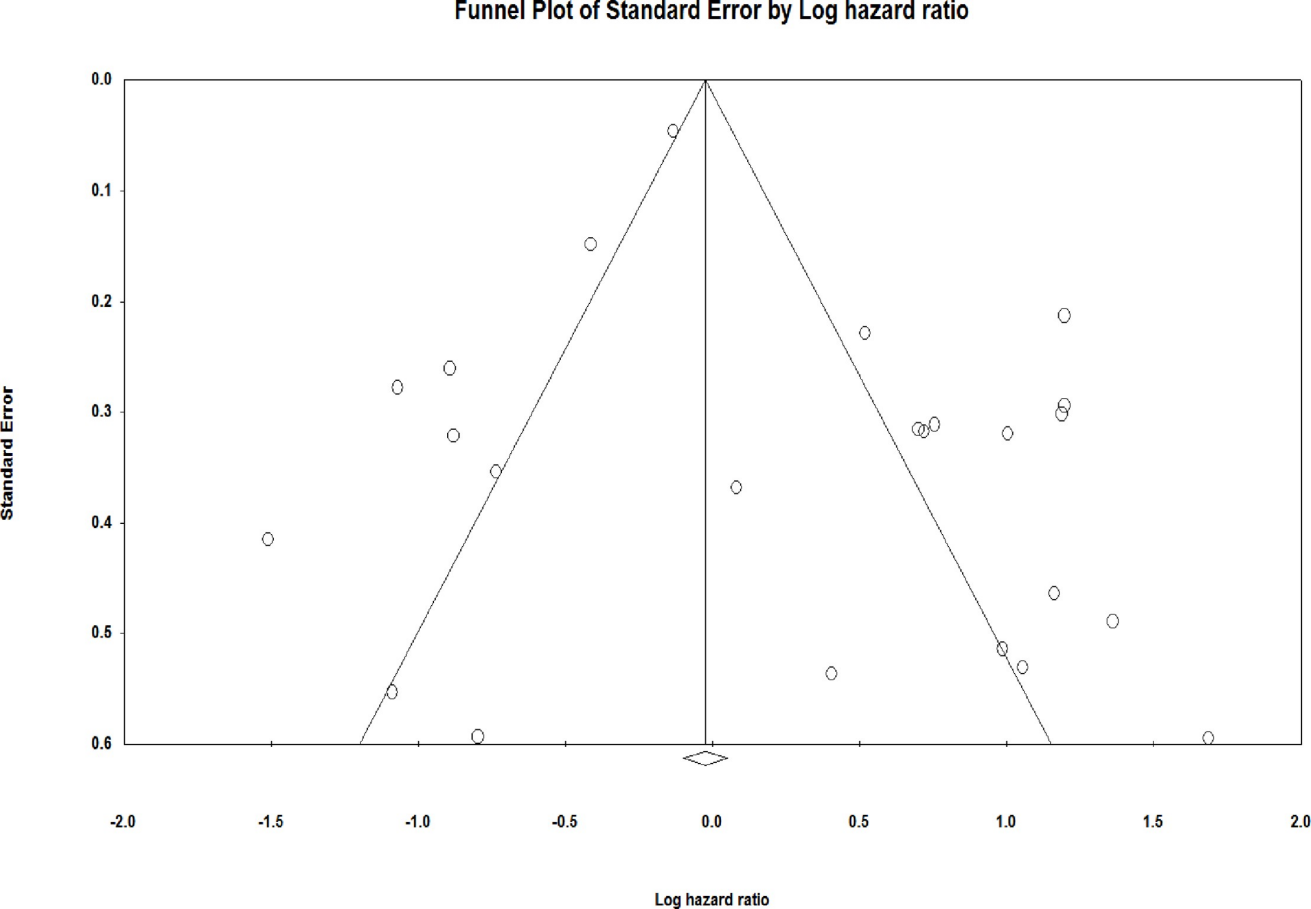
Funnel plot of studies correlating the overall patient survival and miRNA expression. The funnel plot measures the study size standard error and precision on the vertical axis as a function of effect size on the horizontal axis. Dots represent the individual study and most of this area contains regions of high significance, which reveals that publication bias would be represented in the form of asymmetry. This would reflect the fact that smaller studies (which appear toward the bottom) are more likely to be published if they have larger than average effects, which makes them more likely to meet the criterion for statistical significance.

Fig 3 shows that the funnel plot was slightly asymmetric across survival outcome studies. This asymmetry could be associated with small study effects (for example. sampling errors) that might cause publication bias in the results of meta-analyses.

#### Classic fail-safe N

This meta-analysis included data from 24 NPC studies, which yielded a Z-value of 2.91427 and a corresponding 2-tailed p-value of 0.00357. The fail-safe N was 30. This means that we would need to locate and include 30 'null' studies for the combined 2-tailed p-value to exceed 0.050. The fail-safe N was significant; we can be confident that the prognostic effects, while possibly inflated by the exclusion of some studies, was nevertheless not nil. Put another way; there would need to be 2.9 missing studies for every observed study for the effect to be nullified.

#### Orwin fail-safe N

The mean hazard ratio in the new (missing) studies can be a value other than the nil value (currently, it is set to 1). Second, the criterion value was an effect size rather than a p-value. The criterion value must be set between the other two values for the Orwin fail-safe N to be computed. Here, the hazard ratio in observed studies was 0.976 which did not fall between the mean hazard ratio in the missing studies which is 1.000

#### Begg and Mazumdar rank correlation test

In this case, Kendall's tau b (corrected for ties, if any) was 0.01087, with a 1-tailed p-value (recommended) of 0.47034 or a 2-tailed p-value of 0.94068 (based on continuity-corrected normal approximation).

#### Egger's test of the intercept

In this case the intercept (B0) was 1.32016, 95% confidence interval (-0.3424–2.9827), with t = 1.64669, df = 22 The 1-tailed p-value (recommended) was 0.0569, and the 2-tailed p-value was 0.11383.

#### Duval and Tweedie's trim and fill

**Fig 4:**
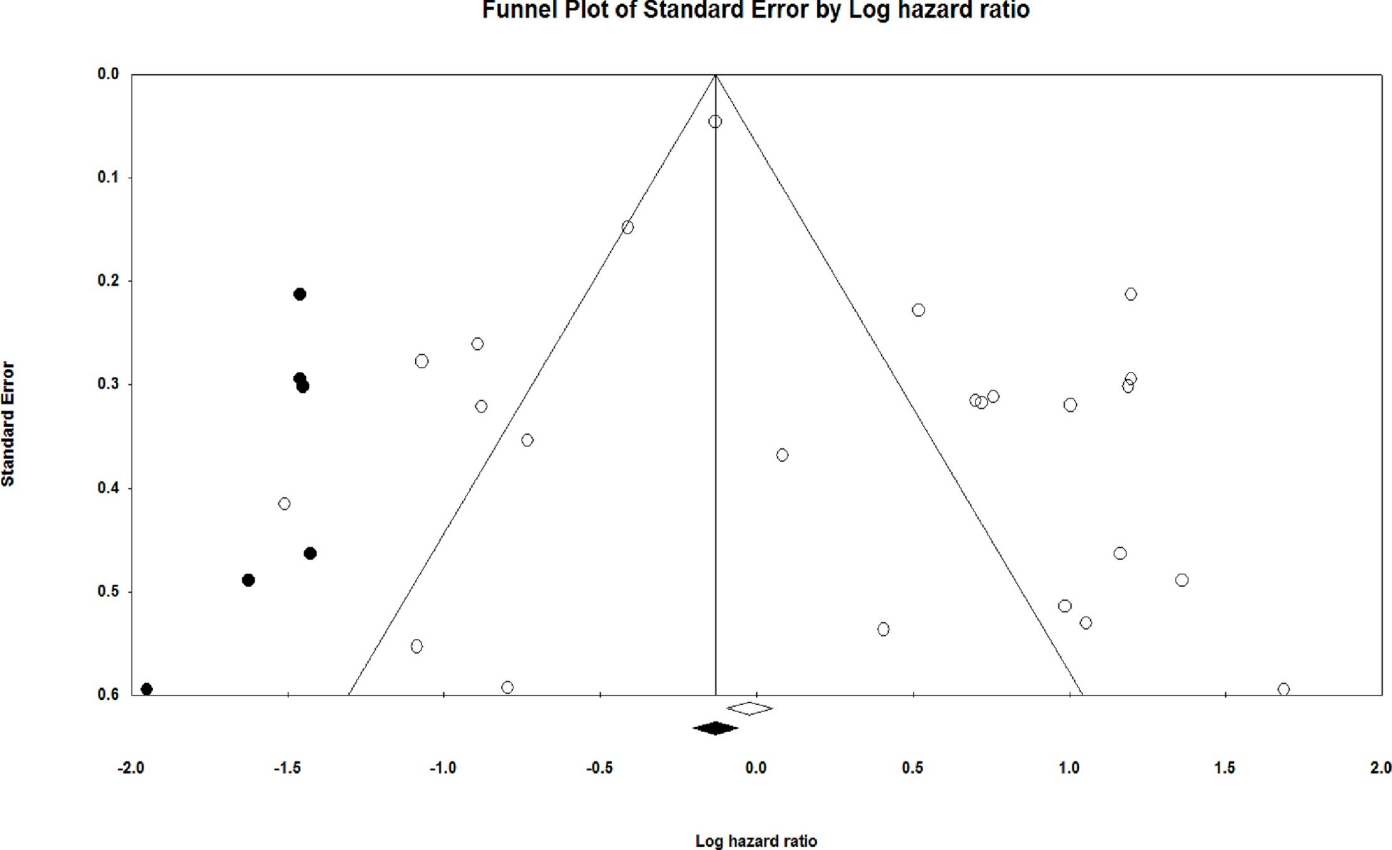
Funnel plot with observed and imputed studies. Large studies appear outside the funnel and tend to cluster on one side of the funnel plot. Smaller studies appear toward the bottom of the graph, and (since there is more sampling variation in effect size estimates in the smaller studies) will be dispersed across a range of values.

Using these parameters, the method suggested that six studies were missing. The funnel plots for trimmed and imputed studies were displayed in Fig 4. Under the fixed effect model, the point estimate and 95% confidence interval for the combined studies was 0.9732 (0.90707–1.05076). Using Trim and Fill the imputed point estimate was 0.8769 (0.81669–0.94078) Under the random effects model, the point estimate and 95% confidence interval for the combined studies was 1.287 (0.947–1.7500). Using Trim and Fill the imputed point estimate was 0.9079 (0.6663–1.237).

### Quality assessment of the selected studies

The criteria and methodological rating are outlined in Table 3. The majority of the studies (15/21) had good quality scores indicating the good methodological quality of included studies and seven studies had satisfactory scores. Though the scores varied between good and satisfactory, the cardinal score (Good- 14 studies, Satisfactory-7 studies) was designated based on the results of HR and 95%CI values which were crucial for this study.

**Table 3:** Quality assessment of the selected studies.

S.No	Criteria	Bad (0–33%)	Satisfactory (33–66%)	Good (67–100%)
**1**	The objective of this paper stated	-	-	22 studies
**2**	Study population clearly specified	-	-	22 studies
**3**	Participation rate of eligible persons at least 50%	-	-	22 studies
**4**	Eligibility criteria	-	-	22 studies
**5**	Sample size justification	-	-	22 studies
**6**	miRNA Exposure assessed before outcome measurement	-	-	22 studies
**7**	Timeframe sufficient for the patients (OS, DFS or MFS)	-	12 studies	11 Studies
**8**	Different levels of the exposure of interest (mode of treatment)	-	Nine studies	13 studies
**9**	Exposure measures and assessment (staging of cancer, TNM)	-	Three studies	19 studies
**10**	Repeated exposure assessment	-	9 studies	13 studies
**11**	Outcome measures (HR, and CI)	-	Seven studies	14 studies
**12**	Blinding of outcome assessors	NA	NA	NA
**13**	Follow-up rate	-	10 studies	12 studies
**14**	Statistical analysis	-	-	22 studies
** **	**Total selected studies**	**0**	**7**	**14**

## Discussion

This systematic review and meta-analysis aims to identify a statistical association between miRNA expression levels and NPC patient survival. As mentioned earlier, a few narrative reviews have hypothesized the possibility of using miRNAs to predict NPC prognosis [5,11,14,15]. The studies included in our systematic review [10,12,32–39,16,40–48,17] have also analysed the variation in miRNA expression based on gender as well as other clinicopathological characteristics. The analysis of NPC patients survival based on gender, through univariate and multivariate analysis, has shown that males have a higher natural predisposition to NPC when compared to females [33] It was observed that the NPC incidence in male NPC patients in China was 2 to 3 fold higher than the female patients [49]. The authors also indicated that high male NPC cases might be explained by factors such as sex hormones and the protective effect of endogenous oestrogen. Additionally, smoking and unsafe work-related exposures may also be considered as risk factors for NPC [50].

The age-specific incidence rates of NPC was also significantly different across various populations [49, 51]. In most low-risk groups, the occurrence rate of NPC was observed to increase with age, similar to that of other epithelial cancers [52]. In contrast, in high-risk groups, the incidence peaks around the ages 50 to 59 years and declines with further advances in age and when nearing death, suggesting the involvement of exposure to carcinogenic agents early in life [53]. Six studies included in our systematic review explored the association between a patient's age and prognosis in NPC. From these studies, it is not evident if the influence of age on patients’ survival was statistically significant.

Jamali and colleagues (2015) investigated the prognostic implications of miRNAs in Head and Neck Squamous Cell Carcinoma (HNSCC) [54]. The findings showed that significantly elevated expressions of miRNA21, miRNA18a and miRNA451 were associated with poor survival in patients with HNSCC. Our findings, despite focusing solely on NPC cases, shared a few interesting overlaps when observing miRNA expression and patient survival. Overexpression of miR21 and decreased expression of miR451 was linked to poor survival and miRNA18a overexpression had a positive impact on patient survival, in both NPC and HNSCC, thereby attributing a possible prognostic utility to these miRNAs. Although NPC was categorised under HNC, the effect of miRNA18a on patient survival in our study differed compared to the findings by Jamali and colleagues where miRNA18a was found to be associated with poor prognosis. Interestingly, miRNA18a and miRNA92a have also been highlighted as miRNA of interest in oesophageal squamous cell carcinoma, where miRNA-18a expression was associated with improvement in both, progression-free survival and overall survival [55]. The impact of miRNA92b on NPC survival was, on the other hand, inconsistent with its effects in Osteosarcoma, as detailed observed in a meta-analysis study, cumulating 25 studies conducted by Kim et al [56].

### Strengths

This study enabled detailed systematic database searches, and comprehensive analytical approach for reporting the most up to date published articles on miRNA prognosticators for NPC. Since the studies in this analysis were obtained from all currently available NPC studies exploring miRNA expression and prognosis, the combined effect size of all selected studies could apply to any future NPC survival studies. The assessment quality score of the included studies revealed that the majority of the studies had a reasonable methodological quality. This is the first systematic review and meta-analysis study which explored the quantitative analysis of miRNAs in NPC. This confirms the guidelines in the “Meta-Analysis Concepts and Applications” a workshop manual by Michael Bornstein.

### Limitations

As some HR and CI values were retrieved from the Kaplan Meier curve, there could be some marginal error as numerical values were not reported explicitly in the full-text of all articles. Liu et 2012 [38] studied the 41 miRNAs together as a high and low-risk score which was included as one study in a forest plot and Liu et al. 2014 [37] studied four miRNAs (miR22, miR572, miR638 & miR1234) combined as high and low-risk group with three separate group (Combined set, training set and validation set). These groups with their HR values were included in forest plot as three individual studies, of the meta-analysis.

Since the total number of studies included in the systematic review and meta-analyses only represented two countries (North Africa and China), the global applicability of this study may also be hampered due to a limitation in currently available published articles. A notable limitation of this study was the small pool of studies taken for meta-analysis. A small set of studies often limits the widespread clinical applicability of the analysis. Therefore, future cohort studies should also consider including a detailed description of multimodal therapy given to NPC patients, which may benefit future endeavours towards an updated systematic review and meta-analysis.

The observation of significant publication bias of this meta-analysis evaluation of 21 studies was revealed by the asymmetrical funnel plot results. To make our results more reliable, we performed Trim and Fill method. More patient cohorts were needed to evaluate in the analysis in order reduce the heterogeneity. This bias also may occur due to some variation in the methods used in each study, as even the same molecular technique can vary between laboratories, due to different kits and reagents, and therefore the threshold levels can vary. Since the number of studies was very less, we could not do any other subgroup analysis. The subgroup effects of miRNAs based on clinicopathological, and other clinical outcome (recurrence) variables were not allowed due to unavailability of HR and CI data in the included studies.

### Future directions

There is currently a lack of knowledge on biomarkers that can reliably provide predictive data for NPC. It is vital to have a robust methodology with larger data sets that might have an impact on the sequence of treatments and determine the aggressiveness of therapies, eventually having a positive effect on survival of NPC patients. It is clear that large-scale, multi-centre prospective clinical studies need to be carried out to determine the role of miRNAs for predicting survival outcomes of NPC patients, especially with regards to adjuvant chemotherapy and there is a high risk of relapse after treatment, even in early-stage diagnosis.

## Conclusions

Our findings support the hypothesis that miRNA plays a significant role in NPC prognosis and the meta-analysis indicates a possible impact of miRNA expression on NPC patient survival outcomes, which may be elaborated upon in future clinical studies. Hence, further rigorous, multi-parameter analysis is required for further validation. However, our study does manage to highlight 65 miRNAs which have potential to function as prognostic markers in NPC, but further comprehensive studies and analyses are essential before the miRNAs reach the stage of therapeutic use in clinical settings against NPC.

## Supporting information

S1 Table PRISMA Checklist for the title “Systematic review and meta-analysis of prognostic microRNA biomarkers for survival outcome in nasopharyngeal carcinoma”. (DOC)
