# Supplementary material for: Systematic review and meta-analysis of prognostic microRNA biomarkers for survival outcome in nasopharyngeal carcinoma
Source: PLoS One. 2019 Feb 8;14(2):e0209760. doi: 10.1371/journal.pone.0209760 (PMC6368411; doi:10.1371/journal.pone.0209760)
Supplement: S1 Table — (DOC) [file pone.0209760.s001.doc]

**Title**: PRISMA Checklist for the title “Systematic review and meta-analysis of prognostic microRNA biomarkers for survival outcome in nasopharyngeal carcinoma”

|  | **Section/topic** |  |  | **#** |  |  | **Checklist item** |  |  | **Reported** |  |  |
| --- | --- | --- | --- | --- | --- | --- | --- | --- | --- | --- | --- | --- |
|  |  |  |  |  |  |  |  |  |  |  |  |  |
|  |  |  |  |  |  |  |  |  |  | **on page #** |  |  |
|  |  |  |  |  |  |  |  |  |  |  |  |  |
|  |  |  |  |  |  |  |  |  |  |  |  |  |
|  | **TITLE** | |  |  |  |  |  |  |  |  |  |  |
|  | Title | | 1 | |  |  | Identify the report as a systematic review, meta-analysis, or both. | |  | 1 |  |  |
|  |  | |  |  |  |  |  |  |  |  |  |  |
|  | **ABSTRACT** | |  |  |  |  |  |  |  |  |  |  |
|  | Structured summary | | 2 | |  |  | Provide a structured summary including, as applicable: background; objectives; data sources; study eligibility criteria, | |  |  |  |  |
|  |  |  |  |  |  |  | participants, and interventions; study appraisal and synthesis methods; results; limitations; conclusions and | |  | 2-3 |  |  |
|  |  |  |  |  |  |  | implications of key findings; systematic review registration number. | |  |  |  |  |
|  |  | |  |  |  |  |  |  |  |  |  |  |
|  | **INTRODUCTION** | |  |  |  |  |  |  |  |  |  |  |
|  |  |  |  |  |  |  |  |  |  |  |  |  |
|  | Rationale | | 3 | |  |  | Describe the rationale for the review in the context of what is already known. | |  | 4-5 |  |  |
|  |  | |  | |  |  |  | |  |  |  |  |
|  | Objectives | | 4 | |  |  | Provide an explicit statement of questions being addressed with reference to participants, interventions, comparisons, | |  |  |  |  |
|  |  |  |  |  |  |  | outcomes, and study design (PICOS). | |  | NA |  |  |
|  |  | |  |  |  |  |  |  |  |  |  |  |
|  | **METHODS** | |  |  |  |  |  |  |  |  |  |  |
|  | Protocol and registration | | 5 | |  |  | Indicate if a review protocol exists, if and where it can be accessed (e.g., Web address), and, if available, provide | |  |  |  |  |
|  |  |  |  |  |  |  | registration information including registration number. | |  | NA |  |  |
|  |  | |  | |  |  |  | |  |  |  |  |
|  | Eligibility criteria | | 6 | |  |  | Specify study characteristics (e.g., PICOS, length of follow-up) and report characteristics (e.g., years considered, | |  | NA |  |  |
|  |  |  |  |  |  |  | language, publication status) used as criteria for eligibility, giving rationale. | |  |  |  |  |
|  |  | |  | |  |  |  | |  |  |  |  |
|  | Information sources | | 7 | |  |  | Describe all information sources (e.g., databases with dates of coverage, contact with study authors to identify | |  | NA |  |  |
|  |  |  |  |  |  |  | additional studies) in the search and date last searched. | |  |  |  |  |
|  |  | |  | |  |  |  | |  |  |  |  |
|  | Search | | 8 | |  |  | Present full electronic search strategy for at least one database, including any limits used, such that it could be | |  |  |  |  |
|  |  |  |  |  |  |  | repeated. | |  | 6 |  |  |
|  |  | |  | |  |  |  | |  |  |  |  |
|  | Study selection | | 9 | |  |  | State the process for selecting studies (i.e., screening, eligibility, included in systematic review, and, if applicable, | |  |  |  |  |
|  |  |  |  |  |  |  | included in the meta-analysis). | |  | 7 |  |  |
|  |  | |  | |  |  |  | |  |  |  |  |
|  | Data collection process | | 10 | |  |  | Describe method of data extraction from reports (e.g., piloted forms, independently, in duplicate) and any processes | |  |  |  |  |
|  |  |  |  |  |  |  | for obtaining and confirming data from investigators. | |  | 7-8 |  |  |
|  |  | |  | |  |  |  | |  |  |  |  |
|  | Data items | | 11 | |  |  | List and define all variables for which data were sought (e.g., PICOS, funding sources) and any assumptions and | |  | 8 |  |  |
|  |  |  |  |  |  |  | simplifications made. | |  |  |  |  |
|  |  | |  | |  |  |  | |  |  |  |  |
|  | Risk of bias in individual | | 12 | |  |  | Describe methods used for assessing risk of bias of individual studies (including specification of whether this was | |  | 8-9 |  |  |
|  | studies | |  |  |  |  | done at the study or outcome level), and how this information is to be used in any data synthesis. | |  |  |  |  |
|  |  | |  | |  |  |  | |  |  |  |  |
|  | Summary measures | | 13 | |  |  | State the principal summary measures (e.g., risk ratio, difference in means). | |  | 8 |  |  |
|  |  | |  | |  |  |  | |  |  |  |  |
|  | Synthesis of results | | 14 | |  |  | Describe the methods of handling data and combining results of studies, if done, including measures of consistency | |  |  |  |  |
|  |  |  |  |  |  |  | (e.g., I^2^) for each meta-analysis. | |  | 8 |  |  |
|  |  |  |  |  |  |  | Page 1 of 2 | |  |  |  |  |


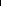

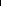

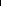

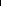

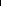

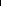

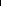

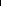


|  | **Section/topic** |  |  | **#** |  |  | **Checklist item** |  |  | **Reported** |  |  |
| --- | --- | --- | --- | --- | --- | --- | --- | --- | --- | --- | --- | --- |
|  |  |  |  |  |  |  |  |  |  |  |  |  |
|  |  |  |  |  |  |  |  |  |  | **on page #** |  |  |
|  |  |  |  |  |  |  |  |  |  |  |  |  |
|  |  |  |  |  |  |  |  |  |  |  |  |  |
|  | Risk of bias across studies | | 15 | |  |  | Specify any assessment of risk of bias that may affect the cumulative evidence (e.g., publication bias, selective | |  |  |  |  |
|  |  |  |  |  |  |  | reporting within studies). | |  | 9-10 |  |  |
|  |  | |  | |  |  |  | |  |  |  |  |
|  | Additional analyses | | 16 | |  |  | Describe methods of additional analyses (e.g., sensitivity or subgroup analyses, meta-regression), if done, indicating | |  |  |  |  |
|  |  |  |  |  |  |  | which were pre-specified. | |  | NA |  |  |
|  |  | |  |  |  |  |  |  |  |  |  |  |
|  | **RESULTS** | |  |  |  |  |  |  |  |  |  |  |
|  | Study selection | | 17 | |  |  | Give numbers of studies screened, assessed for eligibility, and included in the review, with reasons for exclusions at | |  |  |  |  |
|  |  |  |  |  |  |  | each stage, ideally with a flow diagram. | |  | 10-11 |  |  |
|  |  | |  | |  |  |  | |  |  |  |  |
|  | Study characteristics | | 18 | |  |  | For each study, present characteristics for which data were extracted (e.g., study size, PICOS, follow-up period) and | |  |  |  |  |
|  |  |  |  |  |  |  | provide the citations. | |  | 12-15 |  |  |
|  |  | |  | |  |  |  | |  |  |  |  |
|  | Risk of bias within studies | | 19 | |  |  | Present data on risk of bias of each study and, if available, any outcome level assessment (see item 12). | |  | 16 |  |  |
|  |  | |  | |  |  |  | |  |  |  |  |
|  | Results of individual studies | | 20 | |  |  | For all outcomes considered (benefits or harms), present, for each study: (a) simple summary data for each | |  |  |  |  |
|  |  |  |  |  |  |  | intervention group (b) effect estimates and confidence intervals, ideally with a forest plot. | |  | 16-18 |  |  |
|  |  | |  | |  |  |  | |  |  |  |  |
|  | Synthesis of results | | 21 | |  |  | Present results of each meta-analysis done, including confidence intervals and measures of consistency. | |  | 16-18 |  |  |
|  |  | |  | |  |  |  | |  |  |  |  |
|  | Risk of bias across studies | | 22 | |  |  | Present results of any assessment of risk of bias across studies (see Item 15). | |  | 18-20 |  |  |
|  |  | |  | |  |  |  | |  |  |  |  |
|  | Additional analysis | | 23 | |  |  | Give results of additional analyses, if done (e.g., sensitivity or subgroup analyses, meta-regression [see Item 16]). | |  | NA |  |  |
|  |  | |  |  |  |  |  |  |  |  |  |  |
|  | **DISCUSSION** | |  |  |  |  |  |  |  |  |  |  |
|  |  |  |  |  |  |  |  |  |  |  |  |  |
|  | Summary of evidence | | 24 | |  |  | Summarize the main findings including the strength of evidence for each main outcome; consider their relevance to | |  |  |  |  |
|  |  |  |  |  |  |  | key groups (e.g., healthcare providers, users, and policy makers). | |  | 22 |  |  |
|  |  | |  | |  |  |  | |  |  |  |  |
|  | Limitations | | 25 | |  |  | Discuss limitations at study and outcome level (e.g., risk of bias), and at review-level (e.g., incomplete retrieval of | |  |  |  |  |
|  |  |  |  |  |  |  | identified research, reporting bias). | |  | 24-25 |  |  |
|  |  | |  | |  |  |  | |  |  |  |  |
|  | Conclusions | | 26 | |  |  | Provide a general interpretation of the results in the context of other evidence, and implications for future research. | |  | 26 |  |  |
|  |  | |  |  |  |  |  |  |  |  |  |  |
|  | **FUNDING** | |  |  |  |  |  |  |  |  |  |  |
|  | Funding | | 27 | |  |  | Describe sources of funding for the systematic review and other support (e.g., supply of data); role of funders for the | |  | NA |  |  |
|  |  |  |  |  |  |  | systematic review. | |  |  |  |  |
|  |  |  |  |  |  |  |  |  |  |  |  |  |


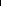


*From:* Moher D, Liberati A, Tetzlaff J, Altman DG, The PRISMA Group (2009). Preferred Reporting Items for Systematic Reviews and Meta-Analyses: The PRISMA Statement. PLoS Med 6(7): e1000097. doi:10.1371/journal.pmed1000097

For more information, visit: **www.prisma-statement.org**.

Page 2 of 2
